# The Dose-Effects of Caffeine on Lower Body Maximal Strength, Muscular Endurance, and Rating of Perceived Exertion in Strength-Trained Females

**DOI:** 10.3390/nu13103342

**Published:** 2021-09-24

**Authors:** Louise Jones, Iona Johnstone, Charlotte Day, Sasha Le Marquer, Andrew T. Hulton

**Affiliations:** Department of Nutritional Sciences, Faculty of Health and Medical Sciences, University of Surrey, Guildford GU2 7XH, UK; joneslm95@googlemail.com (L.J.); iona.johnstone363@gmail.com (I.J.); ca.day@btinternet.com (C.D.); sl00827@surrey.ac.uk (S.L.M.)

**Keywords:** caffeine, strength-trained females, maximal strength, strength endurance

## Abstract

Caffeine supplementation has shown to be an effective ergogenic aid enhancing athletic performance, although limited research within female populations exists. Therefore, the aim of the investigation was to assess the effect of pre-exercise caffeine supplementation on strength performance and muscular endurance in strength-trained females. In a double-blind, randomised, counterbalanced design, fourteen strength-trained females using hormonal contraception consumed either 3 or 6 mg·kg^−1^ BM of caffeine or placebo (PLA). Following supplementation, participants performed a one-repetition maximum (1RM) leg press and repetitions to failure (RF) at 60% of their 1RM. During the RF test, rating of perceived exertion (RPE) was recorded every five repetitions and total volume (TV) lifted was calculated. Repeated measures ANOVA revealed that RF (*p* = 0.010) and TV (*p* = 0.012) attained significance, with pairwise comparisons indicating a significant difference between 3 mg·kg^−1^ BM and placebo for RF (*p* = 0.014), with an effect size of 0.56, and for 6 mg·kg^−1^ BM (*p* = 0.036) compared to the placebo, with an effect size of 0.65. No further significance was observed for 1RM or for RPE, and no difference was observed between caffeine trials. Although no impact on lower body muscular strength was observed, doses of 3 and 6 mg·kg^−1^ BM of caffeine improved lower body muscular endurance in resistance-trained females, which may have a practical application for enhancing resistance training stimuli and improving competitive performance.

## 1. Introduction

Supplementation with caffeine has become increasingly widespread among athletes since its removal from the World Anti-Doping Agency Prohibited List in 2004 [1,2]. Caffeine, a compound commonly found in foods and drinks such as tea, coffee, soft drinks, and chocolate [3], acts as a stimulant thought to have the potential to elicit ergogenic effects through mechanisms including adenosine antagonism, reducing perceived effect, increased motor unit recruitment, and enhancing fat oxidation [4].

It is well established that caffeine works primarily as an adenosine antagonist [5], competing with adenosine to bind to adenosine receptors, preventing parasympathetic effects of adenosine and increasing alertness during exercise [6]. Further, the feeling of increased vigor following caffeine intake reflects an optimised readiness of the state of preparation of an athlete to undertake physical activity, and may contribute to a reduction in perceived effort levels [7]. Indeed, a meta-analysis concluded that 29% of improvements in submaximal exercise may be due to reductions in RPE [8]. Caffeine has also been recognised to enhance motor unit recruitment, which may be a plausible mechanism for improving resistance exercise performance [9]. The original mechanism purported for caffeine was its ability to spare glycogen and augment fatty acid availability and oxidation, leading to greater endurance performance outcomes [10]. However, this theory is commonly refuted as studies have observed improvements in performance with caffeine in absence of increased lipid oxidation and reliance on glycogen [11,12,13].

Performance enhancements are likely due to the potential multifactorial mechanisms that caffeine has frequently been demonstrated to stimulate across many exercise settings and modalities [14]. It is well recognised that pre-exercise caffeine intake can elicit improvements in endurance performance, high-intensity exercise, team sport exercise performance, and cognitive function, with comprehensive reviews conducted that showcase the plethora of research articles highlighting these areas [4,15,16]. Further observations from contemporary reviews by Guest and colleagues [4] and Grgic [17] suggest that caffeine supplementation protocols may be optimal with doses between 3 and 6 mg·kg^−1^ body mass (BM) consumed 30–60 min prior to exercise, although the authors acknowledge that doses as low as 2–3 mg·kg^−1^ may offer comparable ergogenic properties.

Fewer investigations, compared to endurance exercise, have examined caffeine’s effect on resistance exercise, including maximal muscular strength and muscular endurance [18]. Whilst a meta-analysis has stated that caffeine can improve muscular strength and muscular endurance [19], there are still questions and conflict between the effects on gender [17], muscle group (upper vs. lower) and strength qualities [20]. These concerns are exemplified when investigating the effects of caffeine on strength components for female participants/athletes, with Grgic and Del Coso’s [20] recent meta-analysis concluding that future research is required to investigate the effects of caffeine on lower-body muscular endurance and muscular strength. This may stem from the lack of significance illustrated during lower body strength exercise in females [20], as previous suggestions with male participants state that larger muscle groups or lower body muscle groups had a four- to six-fold larger overall effect size compared to the upper body with acute caffeine supplementation [19]. A possible theory is that larger muscle groups have a higher threshold to attain greater muscle activation via an increase in motor unit recruited due to the stimulation of the central nervous system (CNS) [19].

Recent reports have indicated that only 13% of investigations from 362 primary studies on caffeine and exercise included female participants [20], further highlighting the disparity within the research. Within the meta-analysis [20], only one investigation [21] controlled for the menstrual cycle, which has been reported to effect caffeine metabolism, with Romero-Moraleda and colleagues [22] stating that no studies have investigated muscle performance in females and the potential ergogenic effect of caffeine whilst taking oral contraceptives, which requires further investigation. Therefore, the aims of the current investigation were to investigate the effect of pre-exercise caffeine ingestion on lower body maximal strength and muscular endurance in strength-trained females currently taking oral contraception. Secondary aims were to elucidate a dose–response of caffeine intake on these indices of resistance training and to determine the effect of caffeine on RPE during submaximal resistance exercise, as the stimulation of the CNS to reduce RPE has been suggested as a mechanism for the ergogenic effect [8], yet it is unclear if this mechanism holds true for resistance exercise. Based on previous research, it was hypothesised that pre-exercise caffeine ingestion would augment maximal strength and muscular endurance in a dose–response manner. It was further hypothesised that caffeine would reduce RPE during submaximal resistance exercise.

## 2. Materials and Methods

### 2.1. Participants

Fourteen strength-trained females volunteered to be research participants in the investigation (see Table 1 for participant characteristics). Recruitment was conducted by poster placement in local gyms and contacting the university’s strength/power-based sports clubs. The inclusion criteria required that participants were healthy females taking some form of hormonal contraception. “Strength-trained” was defined as performing resistance-training activity at least 3–5 days per week for the 6-month period immediately prior to enrolment in the investigation [23]. Participants were excluded if they were not using hormonal contraception, were not strength-trained, or had any health problems that may have been exacerbated by caffeine or lifting of heavy weights, such as heart palpitations or lower body injury. Participants were fully informed of all testing procedures, purposes, and risks verbally and in writing. Participants completed a Physical Activity Readiness Questionnaire and provided written consent prior to participation in the investigation, which had received favourable ethical opinion from the University of Surrey Ethics Committee and was conducted in accordance with the Ethical Standards in Sport and Exercise Science Research: 2020 Update [24].

### 2.2. Experimental Design

The investigation was a double-blind, placebo-controlled, counterbalanced crossover design. Participants undertook four visits in total, each at least three days apart. This included one familiarisation session and three experimental trials. Participants were randomised to receive 3 mg·kg^−1^ BM caffeine, 6 mg·kg^−1^ BM caffeine, or placebo (PLA) in a counterbalanced order. Testing took place between 08:00 and 12:00, with each session taking place at the same time for each individual. Participants were asked to refrain from vigorous exercise for 24 h, to exclude caffeine for 12 h, and to fast for at least 2 h prior to each session.

### 2.3. Supplementation Protocol

Prior to each of the three trials, participants consumed a drink that contained 50 mL sugar-free squash, 300 mL water and either caffeine powder (Bulk Powders, England, UK) in a dose of 3 mg·kg^−1^ or 6 mg·kg^−1^ BM dissolved in the solution, or PLA containing squash and water only. Participants consumed each drink 30 min prior to commencing the exercise protocol.

### 2.4. Exercise Protocol

The testing protocol was completed on a leg press machine (Hammer Strength, Life Fitness, Rosemont, IL, USA) to determine 1RM and repetitions to failure (RF) at 60% of their 1RM. A recent review concluded that 1RM tests generally have good to excellent test–retest reliability [25]. During the familiarisation session, participants were instructed on correct technique and required depth (starting concentric position ≤90 degrees of knee flexion) on the leg press machine. Prior to testing, participants completed a 10 min warm up consisting of 5 min on a cross-trainer (Life Fitness, Rosemont, IL, USA) and 5 min of lower body stretches. During the familiarisation session, participants worked up to 1RM by completing single sets of 12–15 repetitions, 5 repetitions, 3 repetitions, and 1 repetition. Following this, each set required participants to complete 1 repetition and the weight was increased until failure. Following a 5 min break, the weight was lowered to 60% of achieved 1RM and participants completed as many repetitions as possible until failure to assess muscular endurance.

**Figure 1:**
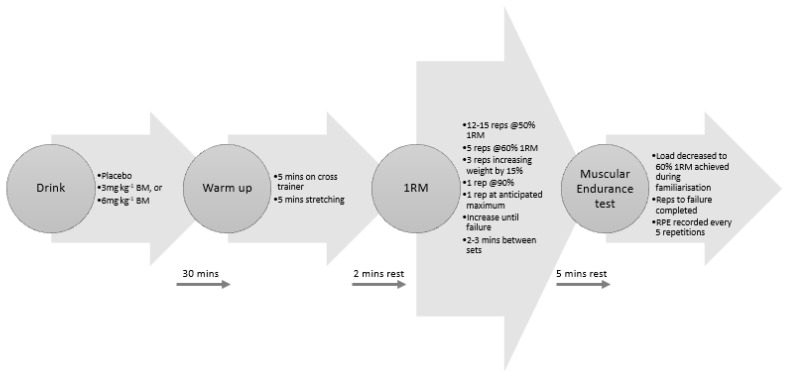
Schematic of the exercise protocol.

Once 1RM was established during the familiarisation, a standardised warm-up was derived for the trial sessions. During the experimental trials, participants completed single warm-up sets of 12–15 repetitions at 50% of 1RM, 5 repetitions at 60% of 1RM, 3 repetitions at 75% of 1RM, 1 repetition at 90% of 1RM, and 1 repetition at 100% of 1RM. The weight was then increased as necessary until participants either perceived that they had reached their maximum or failed a repetition. Participants took 2 min rest intervals between each set. Following 5 min rest, participants completed RF at 60% of the 1RM achieved during the familiarisation. During the RF test, RPE was recorded every 5 repetitions on a scale of 1 to 10 [26]. Total number of repetitions was recorded, and total volume lifted during the RF test was calculated by multiplying total of number of repetitions completed by the load. Participants were given verbal encouragement throughout each trial and all measurements were performed by the same investigator. A simple schematic of the exercise protocol can be seen in Figure 1.

### 2.5. Dietary Standardisation

Participants were asked to maintain their normal dietary patterns throughout their involvement in the investigation. Participants completed a 3-day food diary to include at least one weekend day, reporting all foods and drinks consumed. Habitual caffeine intake was determined by dietary analysis using computer software (Nutritics, Ireland).

### 2.6. Statistical Analysis

Data were analysed using SPSS Version 25 (Chicago, IL, USA). Data normality was assessed using Shapiro–Wilk test, and all variables reported normality. Differences between conditions were analysed by repeated measures analysis of variance (ANOVA) for total volume, RF, 1RM, and RPE at repetitions 5, 10, 15, and 20. In the circumstance that main effects were present, post hoc testing was performed using Holm–Bonferroni adjustments [27]. An a priori power analysis (G*Power, version 3.1.9.7) indicated that a sample size of 18 would allow detection of a significant difference between doses with a high statistical power (1 − β = 0.95: 0.05 = α). Effect size was calculated using Cohen’s d effect size value calculation [28]. All data are reported as mean ± SD unless otherwise stated, and significance was accepted at *p* < 0.05.

## 3. Results

**Figure 2:**
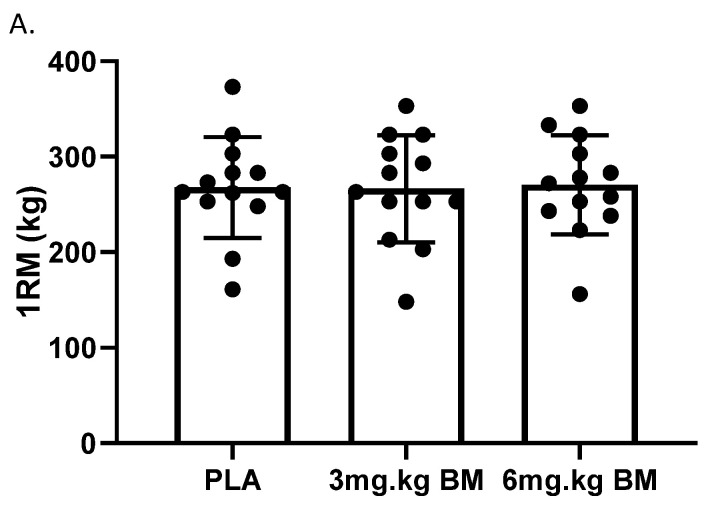

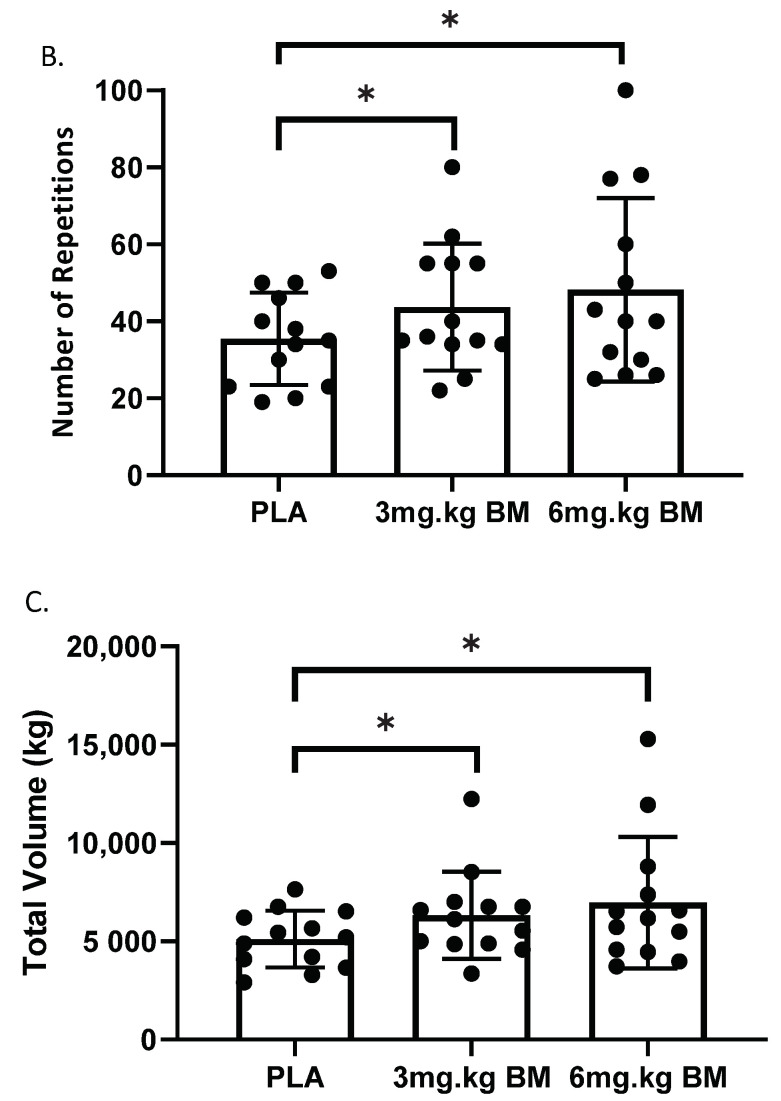
(**A**). 1RM (**B**). Number of reps completed and (**C**). Total volume lifted during the leg press exercise in strength-trained females, with PLA, 3 and 6 mg·kg^−1^ BM. * *p* < 0.05 condition effect.

There was a lack of significance observed between the caffeinated doses and the placebo for 1RM testing data (*p* = 0.731) illustrated in Figure 2A. This analysis is supported by the effect’s sizes of −0.02 and 0.05 for 3 mg·kg^−1^ BM and 6 mg·kg^−1^ BM of caffeine, respectively, identifying no effects of caffeine on muscular strength.

However, repeated measures ANOVA revealed that muscular endurance (RF) (*p* = 0.010) and total weight lifted (TV) (*p* = 0.012) attained significance. Pairwise comparisons were performed to determine the conditions in which the significance was present. This difference held when the Holm–Bonferroni correction [27] was employed for 3 and 6 mg·kg^−1^ BM compared to the placebo. There was a significant difference for the number of repetitions (RF) in the 3 mg·kg^−1^ BM caffeine and placebo trials (*p* = 0.014; mean difference: 8.2 repetitions; 95% CI: 1.6, 14.9 repetitions). Similar findings were observed for the 6 mg·kg^−1^ BM caffeine and placebo trials (*p* = 0.036; mean difference: 12.8 repetitions; 95% CI: 0.7, 24.8 repetitions). Medium effect sizes were observed for both 3 and 6 mg·kg^−1^ BM of caffeine with effect sizes of 0.57 (95% CI: −0.2, 1.3) and 0.68 (95% CI: −0.1, 1.4), respectively. These differences accounted for an increased endurance of 23% for 3 mg·kg^−1^ BM and of 36% for 6 mg·kg^−1^ BM of caffeine. The data for caffeinated vs. non-caffeinated doses for RF and TV can be seen in Figure 2. Data are also presented in Table 2 to support to analysis.

**Figure 3:**
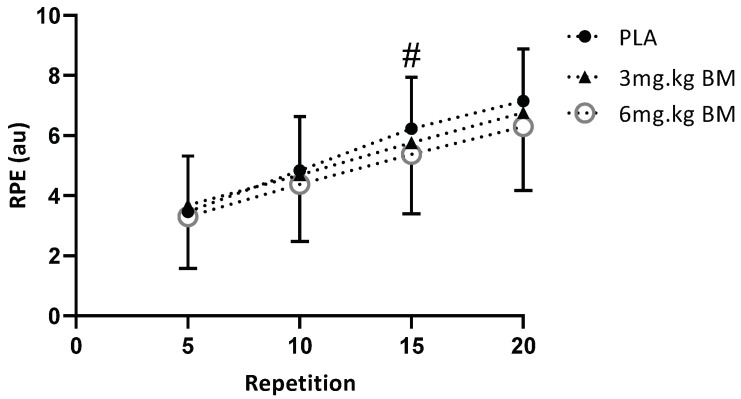
Rating of Perceived Exertion between conditions and repetitions during the endurance performance test at 60% 1RM. # Condition effect identified (*p* < 0.05), but no post hoc difference was statistically significant.

RPE data were only analysed up until rep 20 (Figure 3), as this was the final multiple of 5 that all participants completed. No significant difference was observed for RPE at reps 5 (*p* = 0.414), 10 (*p* = 0.339), or 20 (*p* = 0.183). However, significance was observed at 15 reps (*p* = 0.032), although pairwise comparisons failed to identify any significance, potentially due to a lack of statistical power within the study and a limited sample size. Effect sizes for 3 and 6 mg·kg^−1^ BM during the final RPE measurement were 0.23 and 0.45, illustrating a small to medium effect of caffeine on muscular endurance.

One outlier was removed during the analysis and presentation of data. This amendment resulted in finding a significant post hoc analysis for 6 mg·kg^−1^ BM (*p* = 0.036) compared to the placebo for muscular endurance. The *p* value with the outliner present was *p* = 0.069. This is noteworthy as it strengthens and supports the findings of the effect size and percentage difference. No other difference between results with *n* = 14 or *n* = 13 was identified.

## 4. Discussion

The current investigation has attempted to add to the limited and conflicting literature related to caffeine, females taking oral contraception, and resistance exercise. The main finding of the investigation was that 3 and 6 mg·kg^−1^ BM of caffeine significantly enhanced muscular endurance, which in turn resulted in an increase in total volume lifted compared to the placebo for both conditions. However, muscular strength and RPE were not improved with the addition of caffeine for both 3 and 6 mg·kg^−1^ BM, with similar results reported for all conditions.

The current findings do not fully align with a meta-analysis focusing on caffeine and strength components for females [20]. The meta-analysis concluded that caffeine ingestion is ergogenic for muscular endurance and muscular strength with small effect sizes of 0.25 and 0.18 for endurance and strength, respectively. However, the subgroup analysis data specifically focusing on lower body exercises found no significant difference between caffeine and placebo for muscular strength or endurance [20]. These specific subgroup findings are of interest and concur with the current investigation regarding maximum strength. The current investigation found no link with caffeine and maximum strength with effect sizes of −0.02 and 0.05 for the 3 mg·kg^−1^ BM and 6 mg·kg^−1^ BM doses, respectively. Warren and colleagues’ [19] previous meta-analysis did identify a considerable variation for caffeine and maximal voluntary contraction (MVC), with effect sizes ranging from −0.18 to 2.46, in which the current maximum strength findings fit, but also noted that knee extensor investigations were associated with a six-fold increase in the effect size, equating to a 7% improvement compared to placebo. It is unclear why the strength increases identified in males do not translate to females for the lower body and it was postulated [19] that lower body maximal strength improvements with caffeine may be due to the mechanism of caffeine stimulating the CNS. Indeed, investigations [29,30] have observed muscular activation lower with the knee extensor compared to other muscle groups, reporting 85–95% for knee extensor activation compared to 90–99% for other muscle groups. Therefore, caffeine may be ineffective with muscle groups able to activate to near maximal levels, whereas caffeine can support knee extensors to achieve greater activation via CNS stimulation increasing muscle unit recruitment.

The previously mentioned theory may not support improvements in muscular endurance, although Warren and colleagues [19] did not dismiss the fact that it may be interlinked. The current investigation found muscular endurance improvements with medium effects sizes (3 mg·kg^−1^ BM =0.57; 6 mg·kg^−1^ BM = 0.68). These findings are supported further with studies in males, with effect sizes ranging from small to large (0.18–2.21) [17]. The meta-analysis specific to females [20] found an overall small effect size of 0.25 for endurance improvements, which was non-significant when comparing the lower body only. Although the mechanisms behind the ergogenic effects of caffeine were not examined in the current investigation, it is well established that caffeine can elicit a wide range of physiological effects [4,14]. As well as the previously discussed stimulatory effects on the CNS, it has been proposed that the most likely mechanisms for its efficacy on strength parameters include adenosine antagonism and increased motor unit recruitment. Caffeine can cross the blood–brain barrier and bind to the adenosine receptors in the brain (A1 and A2a), which reduce the inhibitory effects of adenosine and thus elicit exercise performance enhancements [5]. These inhibitory effects may be the cause for the reduction in RPE that has been reported via meta-analysis [8] and suggest that a reduction of almost 6% during steady state exercise may partially account for any performance gains. RPE is commonly reported to be unaltered at exercise termination, which may be suggested as a design flaw within research involving resistance exercise, with the perception of effort throughout the exercise missed. However, the current investigation recorded RPE throughout the endurance task, and although a significant effect was not identified between conditions, small to medium effect sizes were observed for the caffeine conditions (3 mg·kg^−1^ BM = 0.23; 6 mg·kg^−1^ BM = 0.45), with a 5.5–11.8% reduction noted for RPE during the 20th rep. A further mechanism for caffeine is its ability to enhance motor unit recruitment by augmenting the release of calcium from the sarcoplasmic reticulum [31]. This may lead to an enhancement in the force of muscular contraction, leading to an improvement in muscular strength. As caffeine has been shown to enhance motor unit recruitment, it may be expected that caffeine ingestion would improve performance in larger muscles of the lower body, compared to smaller muscles of the upper body as previously stated [19]. There is further support from Black and colleagues [30] that the ergogenic responses are likely to be due to increased motor unit recruitment as they cast doubt on caffeine’s ability to reduce pain and perception at higher intensities despite improved performance. They conclude that the enhanced strength and increased muscle activation could represent the most plausible mechanism in which caffeine exerts it ergogenic effect during resistance exercise.

Although research is increasing with female populations within this domain, the current lack of exploration with regard to females and their response to resistance exercise and/or caffeine is likely due to the hormonal implications associated with strength performance [32] and caffeine metabolism [33]. The effects of the follicular and luteal phases of the menstrual cycle have been shown to affect strength training, with a higher gain in strength during the follicular phase [32]. However, this conflicts with previous information and reports of a lack of significance between phases have been identified with hormonal contraceptive use [34] or without [35] its use. Prior observations have concluded that caffeine metabolism was significantly slower in the luteal phase of the menstrual cycle compared to the follicular phase [33]. This slowed metabolism could lead to a larger accumulation and prolonged effects of caffeine, potentially leading to greater ergogenic effects during the luteal phase [36]. However, an investigation did explore the use of caffeine during the early follicular phase [21], which was selected due to this phase resulting in the lowest variability in ovarian hormones [37] and significant improvements were observed in both strength and endurance. The lack of previous control investigations [23,38,39] may have led to varying rates of metabolism at different points throughout the menstrual cycle, potentially confounding results. In contrast, as stated the current investigation used strength-trained females who were using hormonal contraception, which may have alleviated the chance of individual hormonal fluctuations influencing responses to caffeine [21]. However, whilst the use of hormonal contraception may have reduced fluctuations in ovarian hormones, they may have also delayed the time needed to reach peak plasma caffeine concentration due to a decrease in the cytochrome P-450 enzyme system [40], which is involved in the major pathway for caffeine metabolism [41]. The findings from the current investigation can only be inferred for females using hormonal contraception, and caution should be taken when extrapolating these data to eumenorrheic females. Further research is required, and it may be central to investigate the caffeine performance effects to resistance exercise across the phases to gain further insight.

### 4.1. Suggestions for Future Research

The current literature suggests that caffeine has acute effects on resistance exercise performance [18]. Little research has been conducted to observe chronic effects of long-term pre-exercise caffeine supplementation [16]. The current investigation displays a performance enhancement for caffeine and muscular endurance for the lower dose and higher dose. Theoretically, if caffeine can acutely improve muscular endurance when taken 30 min prior to exercise, this could benefit long-term adaptations to resistance training. By increasing the number of repetitions at a given load, the total volume would be increased without any increase in the perceived effort associated with additional load. Whilst the current investigation only observed total volume lifted in one set, greater accumulation of volume over time may augment muscular hypertrophy [42]. Muscular hypertrophy is one of the primary adaptations to resistance training and is sought after for many athletic populations, especially strength and power athletes such as weightlifters and powerlifters, as it may assist in building strength. To examine the efficacy of caffeine in these sports, future research should investigate its effects on competitive strength athletes. In addition, the use of multi-ingredient pre-workout supplements (MIPS) has gained popularity in recent years [43], with caffeine included as a main ingredient along with other supplements such as creatine, nitic oxide agents, β-alanine and amino acids. A brief review into MIPS concluded that MIPS have promise as an ergogenic aid for active individuals due to their synergistic effect on acute training and performance and may form another aspect of future research.

### 4.2. Practical Implications

A notable finding of the investigation was that the higher caffeine dose did not significantly improve performance further than the lower dose, suggesting lower caffeine doses would be needed to be consumed for the same beneficial effect, potentially limiting the adverse effects associated with higher doses of caffeine [17]. However, a small effect size of 0.22 and a 10% improvement were observed between doses, favouring the higher caffeine dose. Furthermore, the practical significance of these findings is of interest and the addition of both caffeine doses improved endurance beyond the smallest worthwhile change (SWC) [44]. The SWC for the endurance test based on the placebo trial was 2.40 reps, which was clearly met by both caffeine trials. Nonetheless, for the average female in the current investigation, 3 mg·kg^−1^ BM equated to 192 mg of caffeine, which is approximately the amount contained in an average large coffee/latte from a commercial coffee shop [45,46] and not a great deal higher than the habitual intake from the group (110 mg·day^−1^). This suggests that potential improvements could be observed with commercially available coffee. Further research is required on coffee as a source of caffeine for ergogenic effects on resistance-trained females, as this is an accessible and practical way for athletes to consume caffeine prior to exercise and has been shown to be successful in endurance events [47] and resistance-trained males [48].

### 4.3. Strengths and Limitation of the Study

Experimental design had several strengths (double-blind, placebo controlled, repeated measures, crossover design), and by utilizing the leg press to measure strength of a large muscle group, the technical elements of the exercise were removed, which may contrast to the back squat. Further, testing was conducted between 08:00 h and 12:00 h to eliminate any strength differences due to time of day, as strength appears to peak in the evening hours [16,49]. It is evident that the small sample, due to the cessation of testing due to the global COVID-19 pandemic, is a limitation and should be recognised prior to interpretation of findings and extrapolation of results, as should the specific population group. Further, as the blinding of the drinks was not evaluated, this is a major limitation, as identified in previous research [50], and although no statistical difference was observed when analysing the order of test administration, it still warrants highlighting. Only females that were strength-trained and using hormonal contraception were included and, therefore, these results can only be assumed for this population, although as previously stated the timing of peak plasma caffeine concentration needs to be identified. Future research should be conducted on eumenorrheic females and those of different training status. Some research has suggested that the effects of caffeine may differ between trained and untrained individuals, and this may be worth investigating further [51]. Consideration was given for the effect of hormonal contraception, although the type of contraceptive pill (i.e., oestrogen-based or progesterone-based) and timing of phase in the menstrual cycle was not controlled for and should be a focus of future research.

## 5. Conclusions

Caffeine at doses of 3 and 6 mg·kg^−1^ BM improved muscular endurance in strength-trained females using hormonal contraception. Post hoc significance was present only for 6 mg·kg^−1^ BM with the removal of an outlier. Higher quantities of caffeine did not result in further statistical significance in strength endurance but may hold meaningful effects with a small effect size of 0.22 and an increase greater than the SWC. No improvements in maximal strength were observed between conditions nor a reduction in the perception of exertion. Application of the results refer to the lower body and it cannot be assumed that the same results would occur with changes to muscle group, mode, volume, or intensity.

## Figures and Tables

**Table 1:** Participant characteristics.

Age (years)	23.3 ± 3.9 (19.0–30.0; 22.5)
Height (m)	1.64 ± 0.06 (1.53–1.73; 1.65)
Body mass (kg)	64.1 ± 10.4 (50.9–86.6; 61.1)
Body Mass Index (kg/m^2^)	23.6 ± 3.1 (20.1–30.7; 23.1)
Training frequency (sessions·week^−1^)	4.0 ± 1.0 (3.0–5.0; 4.0)
Baseline 1RM (kg)	249.5 ± 48.6 (148–322; 255)
Habitual caffeine intake (mg·day^−1^)	109.7 ± 73.4 (0.0–245.0; 116.5)

Mean ± SD; range and median in parentheses. *n* = 14.1RM, 1 repetition maximum

**Table 2:** Raw and mean data (±SD) for strength and endurance measures for each condition.

Participant	Maximum Strength-1RM (kg)			Muscular Endurance-60% 1RM (*n* of Reps)		
	PLA	3 mg·kg BM	6 mg·kg BM	PLA	3 mg·kg BM	6 mg·kg BM
1	323	323	333	23	36	30
2	253	253	253	23	35	26
3	263	253	283	19	22	26
4	283	303	323	50	80	100
5	303	323	303	20	25	25
6	373	353	353	35	35	32
7	263	283	243	53	55	60
8	193	203	223	50	62	78
9	273	253	258	38	34	40
10	283	293	278	34	40	43
11	248	213	238	30	34	40
12	161	148	156	46	55	50
13	262	263	272	40	55	77
Mean ± SD	267.77 ± 52.86	266.46 ± 56.03	270.46 ± 51.95	35.46 ± 11.98	43.69 ± 16.50	48.23 ± 23.84

## Data Availability

The data presented in this study are openly available in FigShare at https://doi.org/10.6084/m9.figshare.16669267.v1 (accessed on 22 August 2021).
